# Characteristics of intestinal microbiota in preterm infants and the effects of probiotic supplementation on the microbiota

**DOI:** 10.3389/fmicb.2024.1339422

**Published:** 2024-03-07

**Authors:** Sen Yang, Jing He, Jing Shi, Liang Xie, Yang Liu, Ying Xiong, Hanmin Liu

**Affiliations:** ^1^Department of Pediatric Pulmonology and Immunology, West China Second University Hospital, Sichuan University, Chengdu, China; ^2^School of Medical and Life Sciences, Chengdu University of Traditional Chinese Medicine, Chengdu, China; ^3^Department of Pediatrics, The Fifth Peoples Hospital of Chengdu, Chengdu, China; ^4^Key Laboratory of Birth Defects and Related Diseases of Women and Children (Sichuan University), Ministry of Education, Chengdu, China; ^5^NHC Key Laboratory of Chronobiology (Sichuan University), Chengdu, China; ^6^Sichuan Birth Defects Clinical Research Center, West China Second University Hospital, Sichuan University, Chengdu, China

**Keywords:** intestinal microbiota, preterm infants, full-term infants, probiotic, 16s rDNA sequencing

## Abstract

**Objective:**

In this study, we investigated the characteristics of the intestinal microbiota of preterm infants, and then analyzed the effects of probiotics supplementation on intestinal microbiota in preterm infants.

**Methods:**

This study enrolled 64 infants born between 26 and 32 weeks gestational age (GA) and 22 full-term infants. 34 premature infants received oral probiotic supplementation for 28 days. Stool samples were obtained on the first day (D1) and the 28th day (D28) after birth for each infant. Total bacterial DNA was extracted and sequenced using the Illumina MiSeq Sequencing System, specifically targeting the V3-V4 hyper-variable regions of the 16S rDNA gene. The sequencing results were then used to compare and analyze the composition and diversity index of the intestinal microbiota.

**Results:**

There was no significant difference in meconium bacterial colonization rate between premature and full-term infants after birth (*p* > 0.05). At D1, the relative abundance of *Bifidobacterium*, *Bacteroides,* and *Lactobacillus* in the stool of preterm infants was lower than that of full-term infants, and the relative abundance of *Acinetobacter* was higher than that of full-term infants. The Shannon index and Chao1 index of intestinal microbiota in preterm infants are lower than those in full-term infants (*p* < 0.05). Supplementation of probiotics can increase the relative abundance of *Enterococcus* and *Enterobacter*, and reduce the relative abundance of *Escherichia* and *Clostridium* in premature infants. The Chao1 index of intestinal microbiota decreased in preterm infants after probiotic supplementation (*p* < 0.05).

**Conclusion:**

The characteristics of intestinal microbiota in preterm infants differ from those in full-term infants. Probiotic supplementation can reduce the relative abundance of potential pathogenic bacteria and increase the abundance of beneficial microbiota in premature infants.

## Introduction

Preterm birth is defined as childbirth occurring before 37 completed weeks of gestation. An estimated 15 million babies are born prematurely each year on a global scale (Walani, 2020). With the widespread use of respiratory support techniques, nutritional support methods, and anti-infective strategies in neonatal intensive care units, the survival rate of preterm infants has been improved, but complications associated with preterm birth remain the leading cause of mortality in children under 5 years of age (GBD 2015 Child Mortality Collaborators, 2016). Preterm infants with a gestational age of less than 32 weeks have a higher risk of mortality and complications, and these preterm infants are reported to have a survival rate of less than 80% before hospital discharge (Saigal and Doyle, 2008; Ministry of Health Malaysia, 2015). Therefore, appropriate treatment in the early life of premature infants is of great significance to reduce complications and promote the survival rate of premature infants.

The intestinal microecology is gradually established after birth. The intestinal microbiota is composed of bacteria, fungi, archaea, protists, viruses, etc., among which bacteria are the main microbial category constituting the intestinal microbiota, which is called intestinal microbiota (Human Microbiome Project Consortium, 2012). It is currently accepted that the intestinal microbiota consists of approximately 500 to 1,000 distinct species. Many of these species are difficult to culture in a laboratory setting, though innovative techniques are being developed to address this issue (Lewis et al., 2021). There are about 20,000 human genes, whereas there are about 1,000 species of bacteria in the intestines, each with 2,000 genes, for 2 million genes, equivalent to 100 times the total number of human genes (Gilbert et al., 2018).

Therefore, the intestinal microbiota is regarded as an additional organ of the body and has a significant role in human health and disease (Munyaka et al., 2014; Robertson et al., 2019). It is generally believed that the uterus is a sterile environment and the colonization of intestinal microbiota commences after birth. However recent research findings have indicated that bacteria may be present in the placenta, umbilical cord, and amniotic fluid, so bacterial exposure may begin before delivery (Aagaard et al., 2014). However, some studies have pointed out that there is insufficient evidence for the theory of colonization of intestinal microbiota before delivery (Perez-Muñoz et al., 2017). Although the source of initial intestinal microbiota colonization in infants is still controversial, delivery mode, gestational age, feeding mode, and environment are important factors affecting intestinal microbiota colonization in early infants (Vandenplas et al., 2020).

Over the past few years, a plethora of studies have demonstrated that the composition of infant intestinal microbiota exerts an influence on children’s growth and development (Li et al., 2019; Robertson et al., 2019). They have been linked with various diseases such as neonatal necrotizing enterocolitis (NEC; Baranowski and Claud, 2019), childhood obesity (Liu et al., 2021), asthma (Barcik et al., 2020), hypertension (Verhaar et al., 2020), diabetes (Iatcu et al., 2021), and other diseases in adulthood. Previous studies have shown that the colonization of beneficial bacteria in the intestinal microbiota is delayed in preterm infants, while they have a higher number of potentially pathogenic bacteria (Westerbeek et al., 2006). Although clinical studies have shown the potential of probiotics in reducing the incidence of NEC (Duffield and Clarke, 2019), only limited studies have simultaneously conducted longitudinal microbiota profiling to assess the influence of supplementation on the composition of gut microbiota. Nonetheless, interventions aimed at “normalizing” the gut microbiota of preterm infants remain an attractive approach to enhancing health and mitigating the risk of disease (Stewart et al., 2018).

In this study, we took preterm and term infants as our main focus to analyze the characteristics of intestinal microbiota. Additionally, we looked at the effect of intestinal microbiota after probiotics supplementation in premature neonates. The study provides more evidence for the clinical treatment idea of probiotic supplementation in early life to reduce complications and promote long-term health in preterm infants.

## Patients and methods

### Study participants

The subjects used in this study were selected from a patient biobank database by the West China Second University Hospital of Sichuan University. Premature infants who were born and admitted to the neonatal intensive care unit of West China Second Hospital of Sichuan University between December 2019 and May 2021 were enrolled. Inclusion criteria were: preterm infants [gestational age less than 32 weeks or full-term infants (born at 37–42 weeks gestational age)]; appropriate-for-gestational age (with birth weight between the 10th and the 90th centile; Cheng et al., 2020). Exclusion criteria were as follows: infants with congenital malformations; intrauterine growth retardation; neonatal hypoxic–ischemic encephalopathy; immunodeficiency or severe infectious diseases; mother’s antibiotic therapy for more than 3 days within 2 weeks before delivery; the mother or the neonate used probiotics or prebiotics during perinatal period; and the guardian does not agree to participate or withdraws from the study. The following information was obtained from the medical records: duration of ruptured membranes; prenatal antibiotic use; delivery mode; GA; birth weight; gender; antenatal steroid treatment, Apgar scores, Neonatal Resuscitation, feeding, Neonatal sepsis, and so on. The study protocol was approved by the medical ethics committee of the West China Second Hospital of Sichuan University, and written informed consent was obtained from the parents or guardians of the neonates (Yang et al., 2021).

### Probiotic supplementation

34 premature infants received oral probiotic supplementation for 28 days and 30 premature infants did not receive probiotic supplements. Probiotics therapy was given using XFLOR® with a daily dose of 1 capsule/d (China Huaxi Biotech Co. Ltd). Each capsule contains *Lactobacillus rhamnosus* (SGL06) 2.5 billion, *Lactobacillus acidophilus* (SGL11) 2.5 billion, *Lactobacillus reuteri* (SGL01) 1.5 billion, *Bifidobacterium* (SGB03) 500 million, Lactoferrin, 100 mg.

### Sample collection and data processing

Stool samples were obtained from baby diapers using sterile test tubes on both Day 1 (D1) and Day 28 (D28). Following collection, these samples were promptly placed in a − 20°C refrigerator until further processing. Library construction using (the NEB Next Ultra DNA Library Prep Kit) kit. Total bacterial DNA was isolated and then sequenced using the Illumina MiSeq Sequencing System based on the V3-V4 hyper-variable regions of the 16S rDNA gene. The test program was not modified. Subsequently, the sequencing results were used to compare and analyze the composition and the diversity index of the intestinal microbiota such as Shannon and chao1. The sequencing service was conducted by Beijing Novogene Genomics Technology Co. Ltd., based in China.

### Statistical analysis

GraphPad Prisms 9 software was used for chart drawing and data analysis. Continuous variables have been presented as means ± standard deviations, while categorical data were presented as ratios or percentages. Differences in continuous variables were assessed using the non-parametric Wilcoxon rank-sum test and Fisher’s exact tests were used to analyze categorical variables. A value of p less than 0.05 was considered statistically significant.

## Results

### Demographic and clinical information

64 preterm infants and 22 full-term infants were enrolled in this study (Table 1). There are statistical differences in gestational age, birth weight, and Apgar scores between the premature and full-term infant groups (*p*<0.05). There were no significant differences in the incidence of premature rupture of membranes, prenatal antibiotic use, delivery mode, and gender between the preterm and full-term groups. In our single-center study, preterm infants in the probiotic group and not probiotic group were approximately matched by gestational age, sex, delivery method, and sample collection time. There were no significant differences in gestational age, birth weight, delivery mode, gender, the incidence of premature rupture of membranes, use of prenatal antibiotics, antenatal steroid treatment, Apgar scores, Neonatal Resuscitation, feeding, and Neonatal sepsis.

**Table 1:** Clinical characteristics of neonates ( $\bar{x}$ ± s, %).

Clinical characteristics		Full-term group (*n* = 22)	Preterm group (*n* = 30)	Preterm probiotic group (*n* = 34)	Full-term vs. preterm group *p*	Preterm vs. preterm probiotic group *p*
GA (weeks)		39.1 ± 1.0	29.2 ± 1.2	28.7 ± 0.9	0.03	0.05
Birth weight (g)		3,321 ± 186	1,238 ± 170	1,251 ± 202	0.02	0.06
Delivery	Vaginal	12(54.5%)	12(40.0%)	12(35.3%)	0.33	0.76
	Cesarean	10(45.5%)	18(60.0%)	22(64.7%)		
Gender	Male	9(40.9%)	14(46.7%)	15(44.1%)	0.22	0.46
	Female	13(59.1%)	16(53.3%)	19(55.9%)		
Premature rupture of membranes		3(13.6%)	10(33.3%)	11(32.4%)	0.71	0.82
Maternal antibiotics^*^		3(13.6%)	7(23.3%)	13(38.2%)	0.76	0.84
Antenatal steroid treatment		-	25(83.3%)	31(91.2%)	-	0.75
Apgar scores	1 min	9.4 ± 0.9	7.3 ± 2.0	6.8 ± 1.5	<0.001	0.24
	5 min	10 ± 0	8.8 ± 1.2	8.4 ± 0.8	<0.001	0.33
	10 min	10 ± 0	9 ± 1.0	8.7 ± 0.7	0.001	0.32
Neonatal resuscitation		-	29(96.7%)	32(91.4%)	-	0.93
The time of enteral feeding starting		-	2.6 ± 1.5	2.1 ± 1.0	-	0.08
Time of full enteral feeding day		-	21.9 ± 15.2	27.8 ± 18.0	-	0.06
Breastfeeding		22(100%)	13(43.3%)	22(64.7%)	0.75	0.87
Neonatal sepsis		-	6(20%)	12(35.3%)	-	0.58

^*^Mother’s intrapartum antibiotic therapy (duration < 3 days).

### Bacterial colonization of meconium

**Figure 1:**
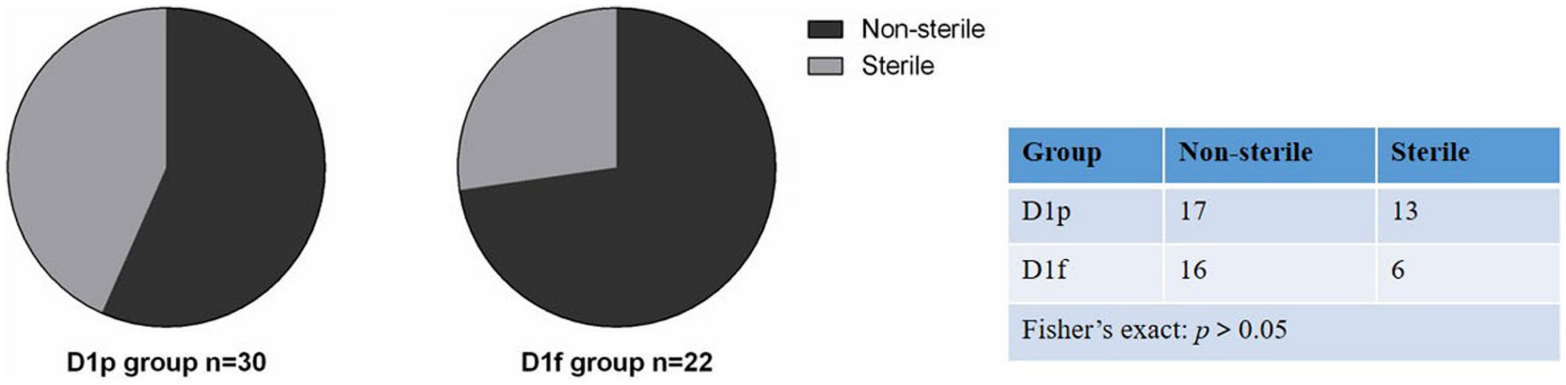
Comparison of bacterial colonization rate in meconium between premature infants and full-term infants.

We collected fecal samples on the first day (D1) and 28th day (D28) after birth. The 16S rRNA gene V3-V4 hypervariable region of all bacteria in meconium was sequenced using Illumina NexSeq high-throughput sequencing technology, and bacteria that could amplify 16S rRNA were considered bacterial colonization. The bacterial colonization rate is the percentage of the number of meconium samples in which 16S rRNA was amplified (Turunen et al., 2021). At D1, 30 fecal samples were collected from infants in the preterm group (D1p), and 16S rDNA was amplified from 17 samples. The colonization rate of meconium was 56.7% (17/30) in preterm infants. 22 meconium samples were collected from infants in the full-term group (D1f), and 16S rDNA was amplified from 16 samples. The colonization rate of meconium was 72.7%(16/22) in term infants. At D28, because some subjects withdrew from the study, 20 fecal samples were collected from the premature group (D28p) and 21 samples from the premature probiotic group (D28p + P), and 16S rDNA was amplified from all the samples. A total of 93 samples were processed for sequencing and sequencing data were successfully obtained from 74 samples (Table 2). There was no significant difference in bacterial colonization rate between the preterm group and the full-term group on the first day after birth (56.7% vs. 72.7%; *p* > 0.05; Figure 1).

**Table 2:** The result of 16S rDNA was amplified from samples.

Group	Number of samples	Non-sterile	Sterile
D1f	22	16	6
D1p	30	17	13
D28p	20	20	0
D28p + P	21	21	0

### Composition and diversity analysis of intestinal microbiota

**Figure 2:**
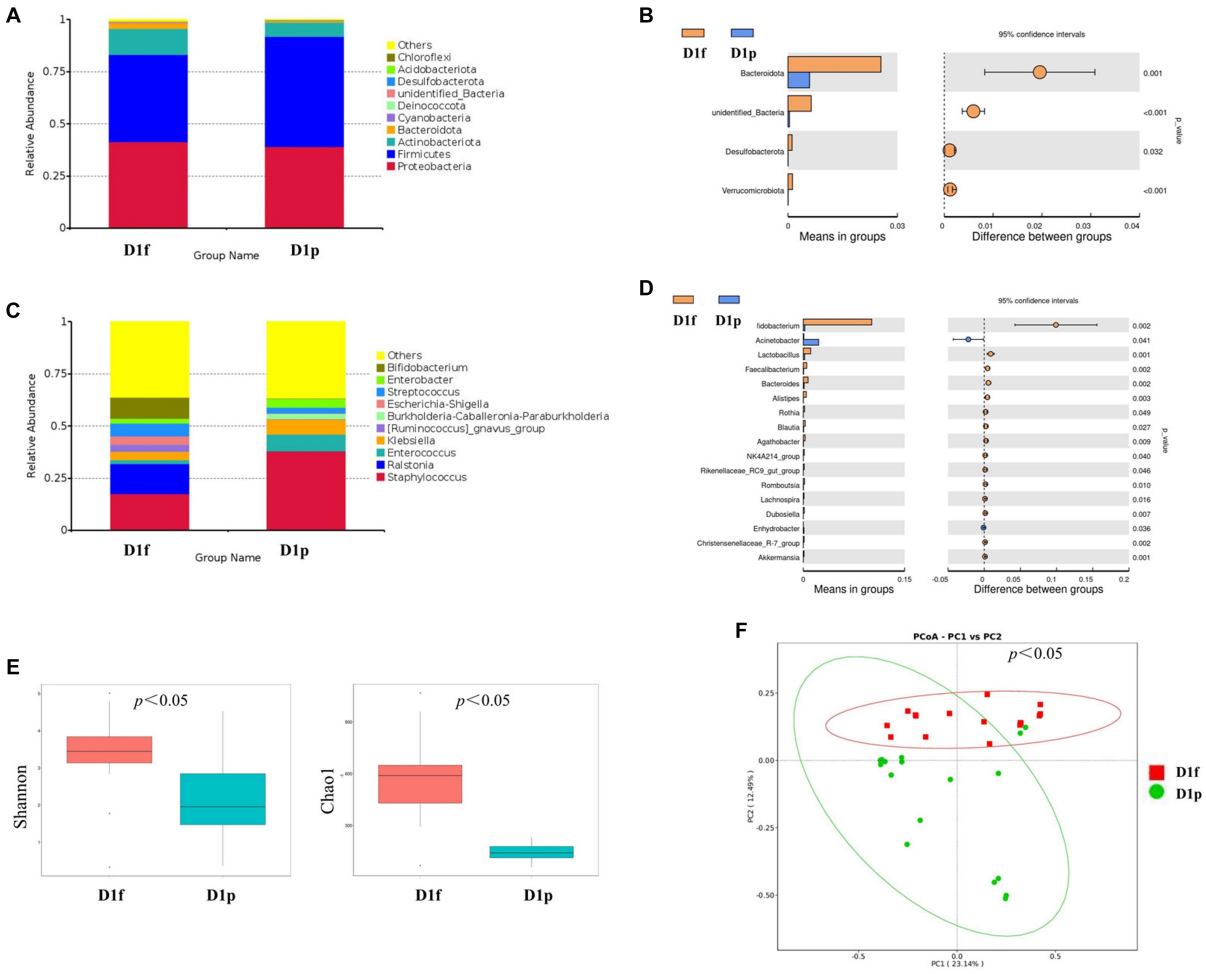
Intestinal microbiota composition and comparison between preterm and full-term infants on the first day after birth. **(A)** Composition of intestinal microbiota at the phylum level. **(B)** Comparison of intestinal microbiota at the phylum level. **(C)** Composition of intestinal microbiota at the genus level. **(D)** Comparison of intestinal microbiota at the genus level. **(E)** Comparison of the microbiota biodiversity, the Shannon index, and Chao1 were shown as estimators. **(F)** PCoA plot based on OTU abundance. Each point represents the intestinal microbiota of a subject.

At the phylum level, the preterm infants (D1p) group has microbiota enriched in *Firmicutes* (52.7%), *Proteobacteria* (39.1%), and *Actinobacteriota* (6.9%), as compared to full-term infants (D1f) group with microbiota dominated by *Firmicutes* (41.9%), *Proteobacteria* (41.4%), and *Actinobacteria* (12.4%; Figure 2A). The D1p group had a lower relative abundance of *Bacteroidetes* than that in the D1f group (0.6% vs. 2.5%; *p* < 0.05). The D1p group also had a lower relative abundance of *Desulfobacterota* and *Verrucomicrobia* (*p* < 0.05; Figure 2B). At the genus level, The D1p group was dominated by *Staphylococcus* (38.0%), *Enterococcus* (8.1%), *Klebsiella* (7.2%), *Enterobacter* (4.3%), and *Streptococcus* (2.8%), as compared to D1f group with microbiota dominated by *Staphylococcus* (17.4%), *Ralstonia* (14.5%), *Bifidobacterium* (10.1%), *Streptococcus* (6.2%), and *Klebsiella* (4.0%; Figure 2C). The relative abundance of *Bifidobacterium* in the D1p was significantly lower compared to the D1f group (0.2% vs. 10.1%; *p*<0.05). Moreover, the relative abundance of *Bacteroides* and *Lactobacillus* in the D1p group was also notably lower than that in the D1f group (*p* < 0.05; Figure 2D).

Both the Shannon index and Chao1 index in the D1p group were found to be lower than those in the D1f group (*p* < 0.05; Figure 2E). To compare the overall composition of the microbiota, PCoA was conducted at the OTU level. The PCoA results revealed a significant difference in microbiota composition between the D1p group and the D1f group (*p* < 0.05; Figure 2F).

### Composition and diversity analysis after probiotic supplementation

**Figure 3:**
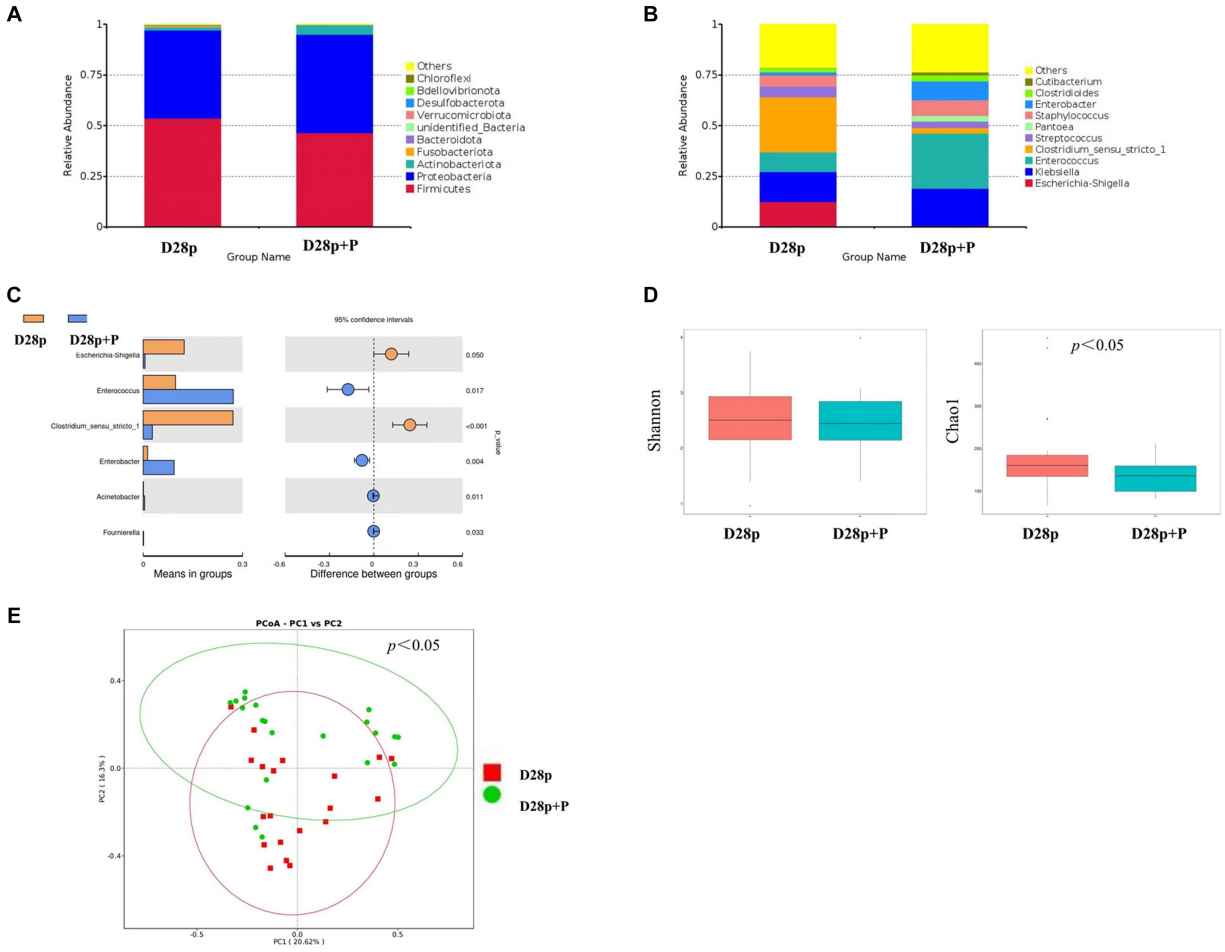
Intestinal microbiota composition and comparison between preterm and full-term infants on the 28th day after birth. **(A)** Composition of intestinal microbiota at the phylum level. **(B)** Composition of intestinal microbiota at the genus level. **(C)** Comparison of intestinal microbiota at the genus level. **(D)** Comparison of the microbiota biodiversity, the Shannon index, and Chao1 were shown as estimators. **(E)** PCoA plot based on OTU abundance. Each point represents the intestinal microbiota of a subject.

At the phylum level, the premature probiotic (D28p + P) group has microbiota enriched in *Proteobacteria* (48.6%), *Firmicutes* (46.5%), and *Actinobacteriota* (4.6%), as compared to premature (D28p) group with microbiota dominated by *Firmicutes* (53.6%), *Proteobacteria* (43.5%), and *Actinobacteria* (1.6%). There was no significant difference in the intestinal microbiota between the two groups at the phylum level (Figure 3A). At the genus level, The D28p + P group was dominated by *Enterococcus* (27.2%), *Klebsiella* (18.4%), *Enterobacter* (9.3%), *Staphylococcus* (7.9%), and *Streptococcus* (3.2%), as compared to D28p group with microbiota dominated by *Clostridium* (27.1%), *Klebsiella* (14.8%), *Escherichia* (12.4%), *Enterococcus* (9.8%), and *Staphylococcus* (5.5%), and *Streptococcus* (5.4%; Figure 3B). The relative abundance of *Enterococcus* and *Enterobacter* in the D28p + P group was higher than that in the D28p group (*p* < 0.05). Moreover, the D28p + P group had a lower relative abundance of *Escherichia* and *Clostridium* compared to the D28p group (*p* < 0.05; Figure 3C).

The Chao1 index of the D28p + P group was significantly lower than that of the D28p (*p* < 0.05; Figure 3D). Furthermore, the PCoA results indicated a significant difference in microbiota composition between the D28p + P group and the D28p group (*p* < 0.05; Figure 3E).

## Discussion

Intestinal microbiota colonization is a complex and dynamic process. The origin of meconium bacteria is not fully understood, but there is growing evidence to suggest that the development of microbiota may commence before birth through microbial transfer across the placental barrier (Ardissone et al., 2014; Rackaityte et al., 2020). In our study, the colonization rate of meconium was 56.7% in preterm infants and 72.7% in term infants. This result is concordant with the theory that the colonization process is initiated prior to the rupture of membranes and birth. While this study suggests that the neonatal gut could be potentially colonized with bacteria before birth, it is important to confirm this finding using a culture-based approach. Furthermore, the precise mechanism of maternal-to-fetus microbiome interactions and their impact on delivery outcomes are yet to be fully understood and require further investigation.

Previous studies have demonstrated that there are significant differences between preterm infants and term infants in their intestinal microbiota characteristics, notably delayed colonization, reduced number of bacterial species, and lower levels of diversity and abundance (Arboleya et al., 2015; Pammi et al., 2017). Furthermore, preterm infants are more susceptible to colonization by facultative anaerobes (*Escherichia*, and *Klebsiella*) that have potential pathogenic effects. They also have reduced levels of commensal strictly anaerobic organisms such as *Bifidobacterium*, *Bacteroides*, and *Clostridium* (Gritz and Bhandari, 2015). In our study, the relative abundance of *bacteroides* in the preterm group was lower than that in the term group. *Bacteroidetes* are metabolically competent and associated with obesity in adults, and their relative abundance increases with age (Bäckhed et al., 2015). We have also found that the relative abundance of *Bifidobacterium* is also lower in the preterm group. *Bifidobacterium* is an important taxon especially during the early days of life, as it is frequently the most prevalent genera in the infant intestines and performs crucial functions in preserving homeostasis. Previous studies have suggested that *Bifidobacterium* colonization occurs in infants with a gestational age of more than 32 weeks (Butel et al., 2007).

When compared to term infants, the intestinal microbiota of preterm infants consistently exhibits reduced microbial diversity (Henderickx et al., 2019). In our study, intestinal microbiota alpha diversity (Shannon index and Chao1 index) of preterm infants was significantly lower compared to full-term infants. We have also found that the composition of the gut microbiota in preterm infants also differs significantly from that of full-term infants. In summary, the preterm group frequently displays atypical patterns of bacterial colonization, dominated by potentially pathogenic bacteria genera such as *Klebsiella*, *Escherichia*, and *Clostridium*. These infants also tend to have lower levels or absence of beneficial genera such as *Bifidobacterium*, which is the dominant genera in the intestines of full-term infants (Dahl et al., 2018; Shao et al., 2019).

Distinct differences can be observed in the intestinal microbiota composition and structure between preterm infants and full-term infants on the first day following birth. Whether the difference in intestinal microbiota is related to the difference in prognosis is still unclear. Since microbiota development is associated with infant gut maturity, especially in very preterm infants, there is a risk of delayed microbiota development and therapeutic intervention may be required. One of the approaches to promote gut microbiota colonization during the early days of life is by oral administration of commensal infant bacteria through probiotic supplementation (Hill et al., 2014). There has been level 1 evidence summarizing the effect of probiotic supplementation: it has been showed to reduce incidences of NEC, sepsis, and all-cause mortality in preterm infants (Dermyshi et al., 2017; Berrington and Fleming, 2019). It is worth noting, despite the positive findings in some studies, one of the largest trials conducted in the UK failed to show benefit from probiotic supplementation (Costeloe et al., 2016). The results of the study showed that probiotic supplementation increased the relative abundance of *Enterococcus*, *Enterobacter,* and *Bifidobacterium* in preterm infants while decreasing the relative abundance of *Escherichia* and *Clostridium*. Hence probiotic supplementation leads to an increase in the relative abundance of beneficial bacteria and a decrease in the relative abundance of harmful bacteria in preterm infants. Although probiotic supplementation increased the relative abundance of beneficial bacteria in the intestine, the actual supplemented bacteria did not become the dominant bacteria in the intestinal microbiota. The possible reason is that the immature intestinal development of preterm infants is selective for the colonization of bacteria, or the dominant bacteria in the intestinal tract of preterm infants (*Staphylococcus* and *Enterococcus*) inhibit the colonization of supplementary bacteria. Earlier studies have suggested that colonization of the gut from probiotic supplementation may vary depending on factors such as the specific strains used, the mode of administration, the dosage used, and the inclusion of prebiotics (Suez et al., 2019). In conclusion, probiotics supplementation improved the composition of intestinal microbiota in preterm infants, increased the relative abundance of beneficial bacteria, and decreased the relative abundance of potential pathogenic bacteria.

To sum up, the characteristics of intestinal microbiota in preterm infants differ from those in full-term infants. Probiotic supplementation has been shown to reduce the relative abundance of potential pathogenic bacteria and increase the relative abundance of beneficial microbiota in premature infants.

## Data availability statement

The data presented in the study are deposited in the repository: https://figshare.com/, available at https://doi.org/10.6084/m9.figshare.25348327.v1.

## Ethics statement

The studies involving humans were approved by Medical Ethics Committee of the West China Second Hospital of Sichuan University. The studies were conducted in accordance with the local legislation and institutional requirements. Written informed consent for participation in this study was provided by the participants’ legal guardians/next of kin.

## Author contributions

SY: Conceptualization, Data curation, Formal analysis, Funding acquisition, Investigation, Methodology, Project administration, Resources, Software, Supervision, Validation, Visualization, Writing – original draft, Writing – review & editing. JH: Conceptualization, Data curation, Formal analysis, Funding acquisition, Investigation, Methodology, Project administration, Resources, Software, Supervision, Validation, Visualization, Writing – original draft, Writing – review & editing. JS: Writing – original draft, Writing – review & editing. LX: Writing – review & editing. YL: Writing – review & editing. YX: Funding acquisition, Writing – review & editing. HL: Funding acquisition, Writing – review & editing.
