# Supplementary material for: Characteristics of intestinal microbiota in preterm infants and the effects of probiotic supplementation on the microbiota
Source: Front Microbiol. 2024 Mar 7;15:1339422. doi: 10.3389/fmicb.2024.1339422 (PMC10956575; doi:10.3389/fmicb.2024.1339422)
Supplement: Supplementary file 1 [file Data_Sheet_1.ZIP › D1ú¿relative abundanceú⌐/top_otu_table_dfdp01_10_genus_group.tar.gz!/top_otu_table_dfdp01_10_genus_group.result/top_otu_table_dfdp01_10_genus_group_no_others.pdf]

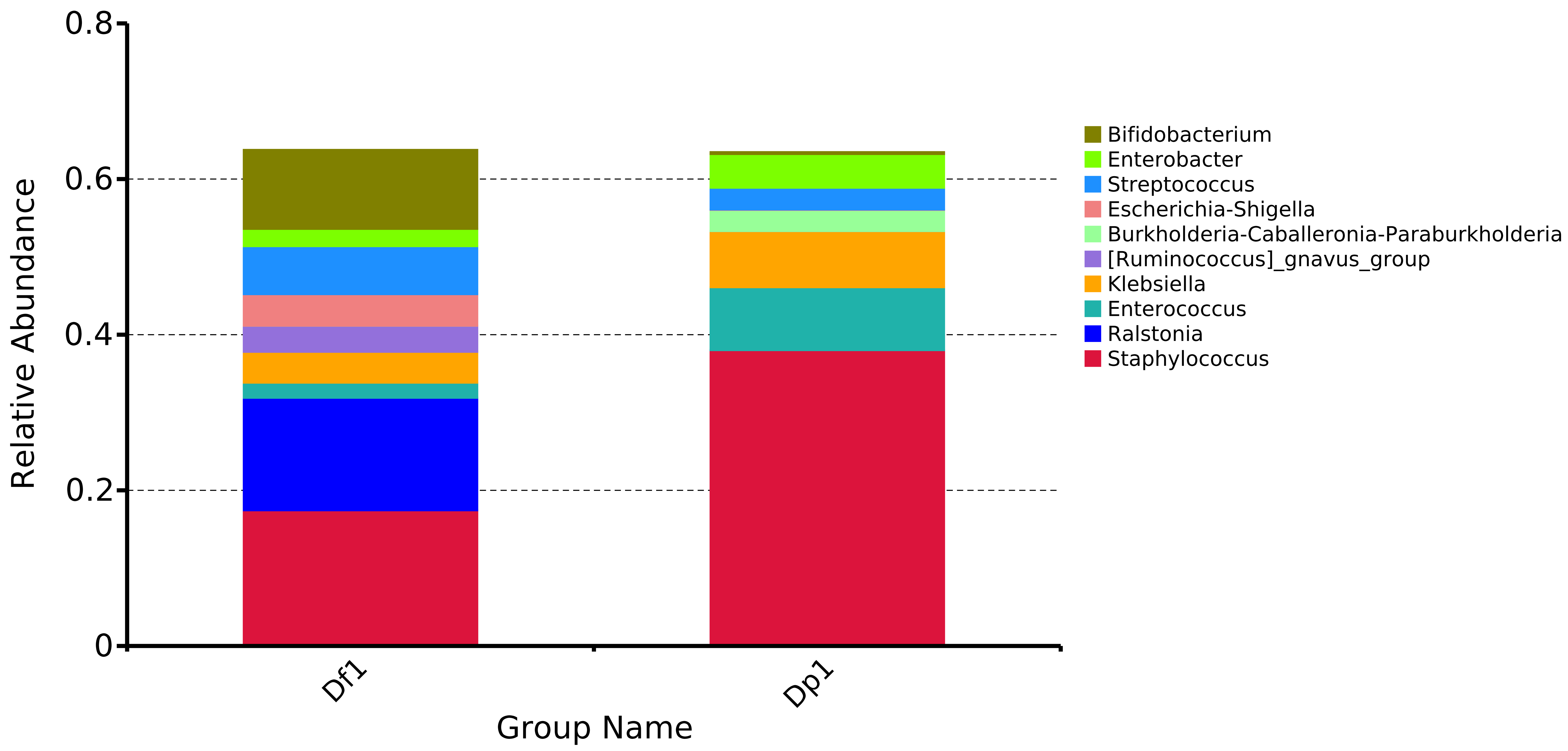

0.8
0.6
0.4
0.2
0
Relative Abundance
Df1
Dp1
Group Name
Bifidobacterium
Enterobacter
Streptococcus
Escherichia-Shigella
Burkholderia-Caballeronia-Paraburkholderia
[Ruminococcus]_gnavus_group
Klebsiella
Enterococcus
Ralstonia
Staphylococcus
