# Supplementary material for: Characteristics of intestinal microbiota in preterm infants and the effects of probiotic supplementation on the microbiota
Source: Front Microbiol. 2024 Mar 7;15:1339422. doi: 10.3389/fmicb.2024.1339422 (PMC10956575; doi:10.3389/fmicb.2024.1339422)
Supplement: Supplementary file 1 [file Data_Sheet_1.ZIP › D1ú¿relative abundanceú⌐/top_otu_table_dfdp01_10_phylum_group.tar.gz!/top_otu_table_dfdp01_10_phylum_group.result/top_otu_table_dfdp01_10_phylum_group.pdf]

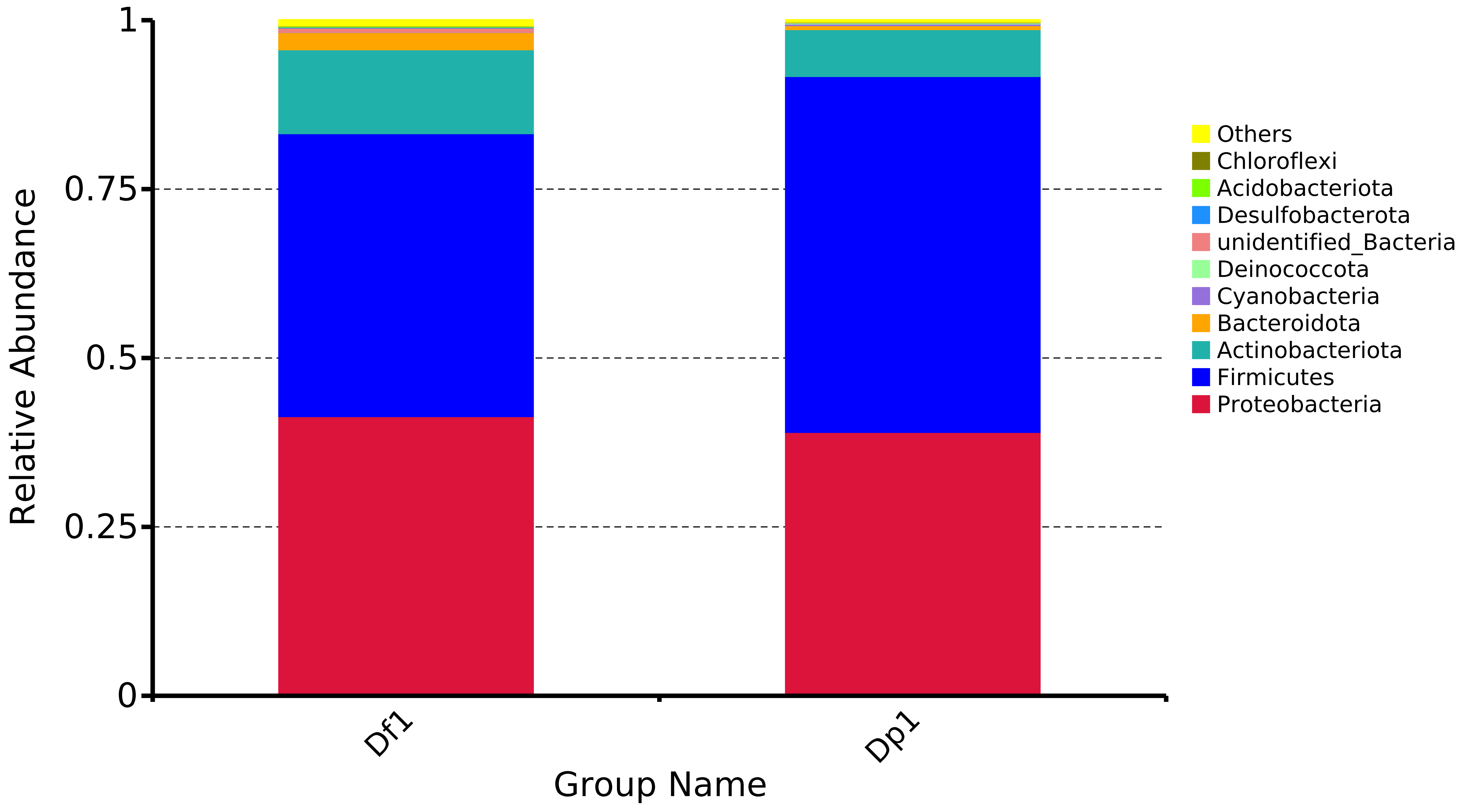

Relative Abundance
1
0.75
0.5
0.25
0
Df1
Dp1
Group Name
Others
Chloroflexi
Acidobacteriota
Desulfobacterota
unidentified_Bacteria
Deinococcota
Cyanobacteria
Bacteroidota
Actinobacteriota
Firmicutes
Proteobacteria
