# Supplementary material for: Characteristics of intestinal microbiota in preterm infants and the effects of probiotic supplementation on the microbiota
Source: Front Microbiol. 2024 Mar 7;15:1339422. doi: 10.3389/fmicb.2024.1339422 (PMC10956575; doi:10.3389/fmicb.2024.1339422)
Supplement: Supplementary file 2 [file Data_Sheet_2.ZIP › D28ú¿relative abundanceú⌐/top_otu_table_T28ysj_10_genus_group.tar.gz!/top_otu_table_T28ysj_10_genus_group.result/top_otu_table_T28ysj_10_genus_group.pdf]

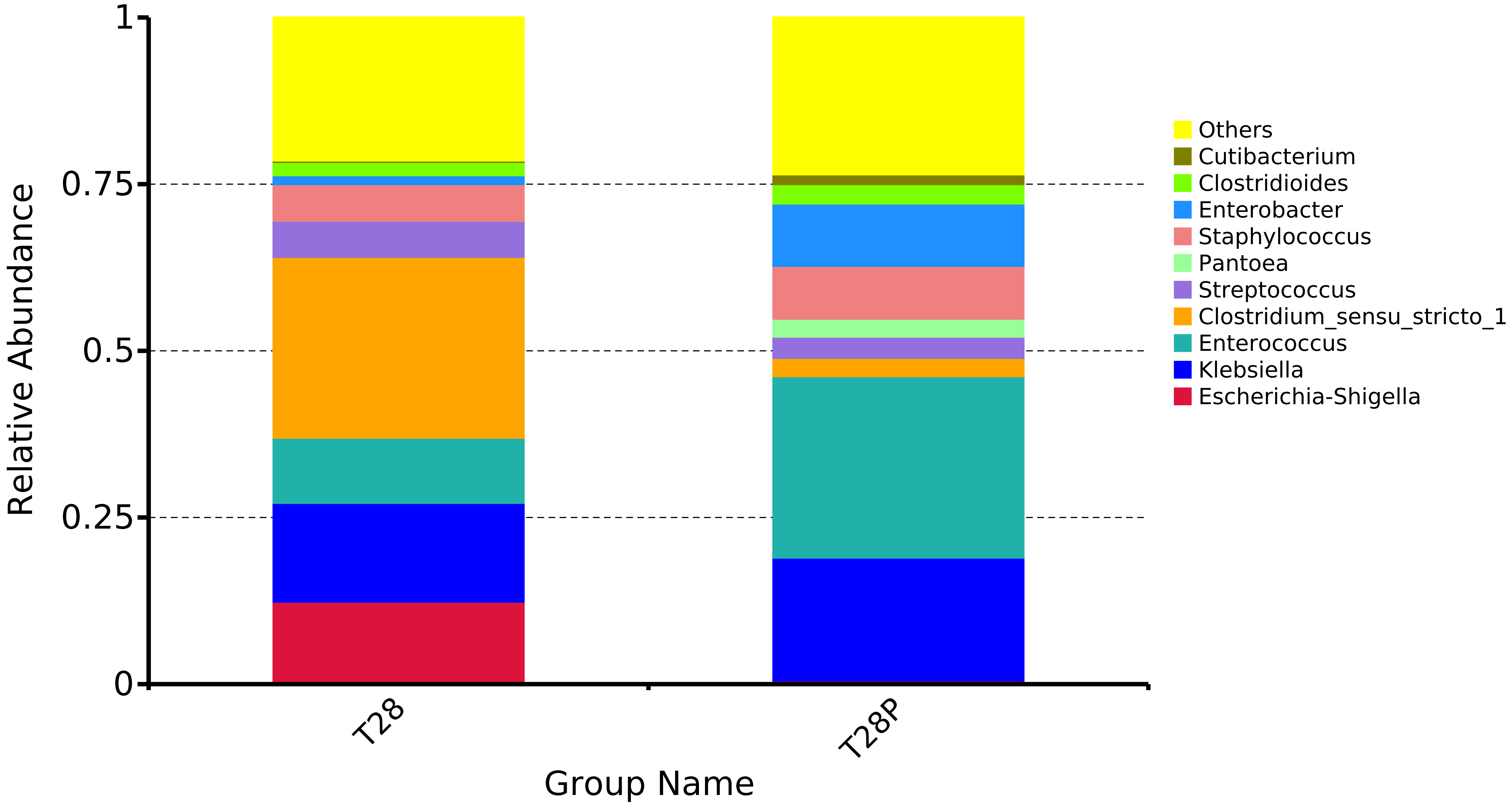

Relative Abundance
1
0.75
0.5
0.25
0
T28
T28P
Group Name
Others
Cutibacterium
Clostridioides
Enterobacter
Staphylococcus
Pantoea
Streptococcus
Clostridium_sensu_stricto_1
Enterococcus
Klebsiella
Escherichia-Shigella
